# Oscillatory autophagy induction is enabled by an updated AMPK-ULK1 regulatory wiring

**DOI:** 10.1371/journal.pone.0313302

**Published:** 2024-12-26

**Authors:** Orsolya Kapuy, Marianna Holczer, Luca Csabai, Tamás Korcsmáros

**Affiliations:** 1 Department of Molecular Biology, Institute of Biochemistry and Molecular Biology, Semmelweis University, Budapest, Hungary; 2 Department of Genetics, ELTE Eötvös Loránd University, Budapest, Hungary; 3 Department of Metabolism, Digestion and Reproduction, Imperial College London, London, United Kingdom; 4 Quadram Institute, Norwich Research Park, Norwich, United Kingdom; Manipal Academy of Higher Education, INDIA

## Abstract

Autophagy-dependent survival relies on a crucial oscillatory response during cellular stress. Although oscillatory behaviour is typically associated with processes like the cell cycle or circadian rhythm, emerging experimental and theoretical evidence suggests that such periodic dynamics may explain conflicting experimental results in autophagy research. In this study, we demonstrate that oscillatory behaviour in the regulation of the non-selective, stress-induced macroautophagy arises from a series of interlinked negative and positive feedback loops within the mTORC1-AMPK-ULK1 regulatory triangle. While many of these interactions have been known for decades, recent discoveries have revealed how mTORC1, AMPK, and ULK1 are truly interconnected. Although these new findings initially appeared contradictory to established models, additional experiments and our systems biology analysis clarify the updated regulatory structure. Through computational modelling of the autophagy oscillatory response, we show how this regulatory network governs autophagy induction. Our results not only reconcile previous conflicting experimental observations but also offer insights for refining autophagy regulation and advancing understanding of its mechanisms of action.

## Introduction

A fundamental property of cellular systems is their ability to form proper answers to external and internal stimuli. Answers can be reversible, irreversible or even periodically repeating according to the stimuli. If the cell is able to return into its original homeostatic state the stimulus is reversible, however if it goes to a new stable state the response is called irreversible [1]. The circadian rhythm and the cell cycle regulatory network can even generate a periodic characteristic of the response mechanism due to the presence of a negative feedback loop in the control network [2–4]. Choosing between life and death is traditionally known as an irreversible response providing a clear directionality of the process. For example, excessive level of cellular stress turns on the suicide cascade of apoptotic cell death. Interestingly, autophagy-dependent self-cannibalism of a cell has also been introduced as a kind of cell death process [1, 5]. However, in the last decades it became evident that it has an important role in cell survival by degrading the damaged or unnecessary components of the cell during cellular stress [6, 7].

Cellular homeostasis is tightly controlled by both mammalian target of rapamycin complex 1 (mTORC1) and 5′ adenosine monophosphate-activated protein kinase (AMPK) [8–12]. While mTORC1 is the master regulator of cellular growth and metabolism [9], AMPK is crucial to sense the proper AMP/ATP ratio in the cell [8]. Both have a key role in regulating the induction of the early stage of non-selective stress induced macroautophagy/autophagy via one of the most important elements of the autophagy induction complex, called unc-51-like kinase 1 (ULK1) [13, 14]. While mTORC1 is able to down-regulate ULK1 under physiological conditions, AMPK has a positive effect on ULK1 upon various stress events (such as food deprivation) [13].

Although traditionally AMPK-dependent phosphorylation sites on ULK1 were thought to be generating a positive effect on ULK1 (see S1 Table), Ji-Man Park et al. have redefined the role of AMPK in autophagy induction by claiming that AMPK has a negative effect on ULK1 via phosphorylating it on its Ser556 residue [15]. Besides, Kazyken et al. have also shown that absence of AMPK increased ULK1 signaling, while mTORC1 activity positively controlled the ability of AMPK to phosphorylate the Ser556 residue of ULK1 further confirming that AMPK has a direct negative effect on ULK1 [16]. A recent publication by Yu-Lin Li has also suggested that AMPK is able to inhibit autophagy supposing that the dual role of AMPK during activation of autophagy [17].

Consequently, it has become kinetically doubtful that autophagy induction itself is an irreversible stress response. Using previous scientific results, we show here that autophagy is indeed a periodically inducible process and that helps cell survival. Here, we also explain recently published, often contradictory results found in the literature, and clarify their contradictions using systems biology modelling.

## Mathematical models and methods

### Mathematical modelling

Ordinary differential equations (ODEs) are used to describe the temporal variation of biological control networks. The equation of the members of the network consists of two parts: an activation (e.g. post-translational modification) and the inactivation of the protein. Mass action kinetics or Michaelis-Menten kinetics are used to describe protein activation and inactivation. Once the set of non-linear ODEs is solved, relative protein concentrations/activities (time courses) can be monitored over time. The qualitative features of the dynamic system were investigated by generating signal-response curves.

Our model is based on the relationships between mTORC1, autophagy and autophagy inducers (ULK1 and AMPK). An extra protein (PROT) has been added to the model to ensure that living systems are described as accurately as possible. The parameter values and the detailed system of equations can be found in the Appendices A (Tables A1 and A2) and B (Tables B1 and B2) in S1 File.

In this work, the time course and signal response curves were computed numerically using XPP-AUT (freely available from https://sites.pitt.edu/~phase/bard/bardware/xpp/xpp.html). The rate constants (k) have the dimension of relative (time unit)^−1^ and Michaelis constants (J) are dimensionless. The protein activities are given in arbitrary units (a.u).

### Network analysis methods

Core, direct and additional protein regulatory layers were downloaded from the AutophagyNet database https://autophagynet.org accessed on 14 March 2024 [18]. From this dataset, interactors were selected that met the defined criteria: (1) getting induced by ULK1 and (2) having a positive effect on AMPK.

## Results and discussion

### A mechanical model of the dynamic characteristic of autophagy induction

**Fig 1:**
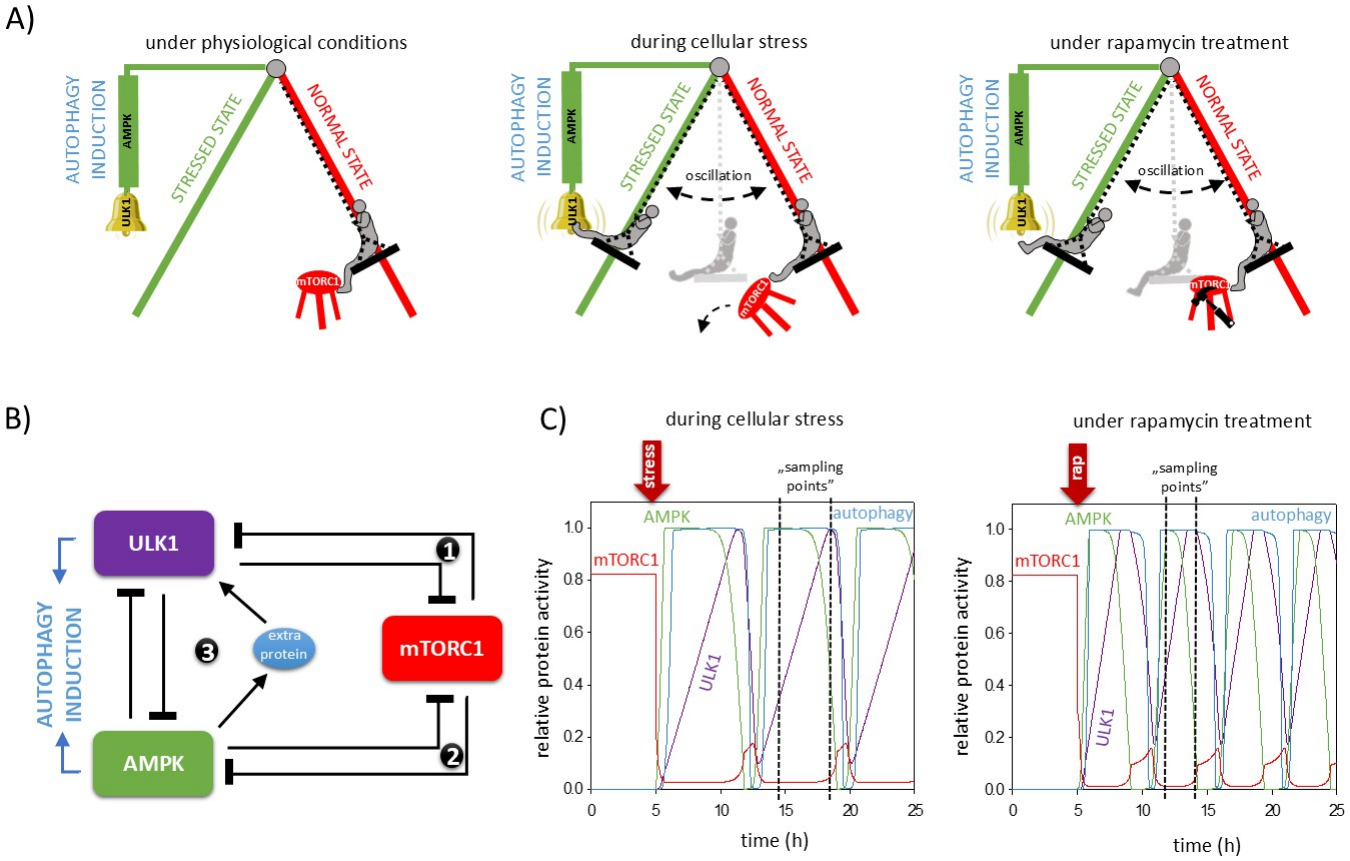
Re-wiring the control network of mTORC1-AMPK-ULK1 regulatory triangle. (**A**) A human-powered swing is a good metaphor of the oscillatory characteristic of cellular stress-induced autophagy. (**panel left**) At normal conditions the swing cannot move because the human is propped up on the footstool with their legs (representing the presence of mTORC1). (**middle panel**) Upon cellular stress, the human leg is pulled up so that mTORC1 can no longer prevent the swing from starting. AMPK and ULK1 get activated, and if both are present (the swing is fully out of position), the bell rings symbolizing the induction of autophagy. Since the swing cannot stop in the autophagy state, it starts to oscillate. (**panel right**) At rapamycin treatment the footstool disappears (as there is no mTORC1), the person can no longer support himself with his legs and the swing swings out to the autophagy position, and the system starts to oscillate. (**B**) The simple wiring diagram of autophagy induction upon cellular stress. Dashed lines show how the molecules can influence each other. Blocked end lines denote inhibition. Numbers refer to the negative and double negative feedback loops of the control network. (**C**) The temporal dynamics is simulated (**panel left**) upon cellular stress (stress1 = 0.25, stress = 0.75) or (**panel right**) rapamycin treatment (mTORC1T = 0.4). The relative activity of mTOR, AMPK, ULK1 and autophagy markers is shown.

To illustrate the idea behind the periodic regulation of autophagy induction, let us use a simple mechanical metaphor built from a human-powered swing and a footstool (Fig 1A). The two legs of the swing stand represent the normal and stressed stated (red and green legs), respectively. The autophagy induced by AMPK and ULK1 in this metaphor is a little bell waiting to be rung by the rocking person, and this is the signal for the activation of the process. However, the presence of a footstool denotes the high mTORC1 level in the cell, which ensures that the rocker cannot swing. As a result, the rocker does not reach the bell thus preventing the autophagy process from being triggered under normal conditions (Fig 1A, upper panel). Cellular stress is symbolized by the rocker stretching its legs and pushing the footstool. Thus, the footstool falls down (decreasing mTORC1 level) and it can no longer prevent swinging the swing to the stressed state in which the rocker can ring the bell referring to the induction of AMPK and ULK1-dependent autophagy. Although the rocker swings out into the stressed state, but does not stay there, as it swings back and forth between the two states (i.e. stressed and normal state). This movement of the rocker generates an oscillatory motion in the system which means that the autophagy bell will only ring periodically and not continuously. The amount of mTORC1 never increases back enough to stop the oscillation of autophagy (Fig 1A, middle panel). In the mechanical metaphor, if someone destroys the footstool with a hammer, it is equivalent to being under mTORC1 inhibition (e.g. during rapamycin treatment). In this case, the rocker does not need to kick up the stool, but easily swings off the swing and oscillates between the stressed and normal states resulting the repetitive ringing of the bell symbolizing the periodic activation of autophagy induction (Fig 1A, lower panel). Until someone puts a stool back to its original position (i.e. mTORC1 is inactive again), the swing will remain in periodic repetitive motion. However, if the rocker does not propel itself on the swing, the movement of the swing will eventually decelerate and then stop, which corresponds to a state of cell death.

### Re-wiring the control network of mTORC1-AMPK-ULK1 regulatory triangle

In order to build up a network to periodically regulate autophagy induction, we compiled experimentally determined published data on the possible connections between mTORC1, AMPK and ULK1 and their sign (positive or negative). A detailed table is attached to the study, which includes both the cells and cell lines used, the stressors used, the treatment times and concentrations and their outcomes (S1 Table).

Based on the literature, we were able to generate a novel regulatory triangle of AMPK-mTORC1-ULK1 (Fig 1B). We claim that AMPK can inhibit mTORC1, meanwhile mTORC1 also has a negative effect on it generating a double negative feedback loop in the control network (see “number 1” in Fig 1B). Besides, a mutual antagonism between ULK1 and mTORC1 is also observed in the control network (see “number 2” in Fig 1B). Due to several identified AMPK targets (called “extra protein” in Fig 1B), we assume that AMPK has a delayed positive effect on ULK1. It is well-known that ULK1 has a negative effect on AMPK, resulting in a ULK1 –| AMPK -> ULK1 negative feedback loop (see “number 3” in Fig 1B). These connections confirm that for autophagy induction, both ULK1 and AMPK are essential, meanwhile mTORC1 inhibits the process.

Taking into account the novel scientific data of Ji-Man Park et al. [15], AMPK is not only able to promote autophagy induction, but it also has a negative effect on the process. Meanwhile the effect of ULK1 kinase is also dual on the self-cannibalism, as it not only directly inhibits AMPK, but also positively affects it via mTORC1 (Fig 1B). These regulatory connections suggest that both ULK1 and AMPK have a dual role, and that this is the key to fine-tuning autophagy induction. When AMPK directly inhibits ULK1, due to the already present ULK1–| AMPK connection a mutual antagonism gets formed between ULK1 and AMPK in the control system. This double negative feedback loop combined with the delayed AMPK-ULK1 negative feedback loop results in a so-called amplified negative feedback loop (see “number 3” in Fig 1B). Our network motif consists of a three-component negative feedback loop (AMPK -> extra protein -> ULK1 –| AMPK) suggesting an oscillatory characteristic, meanwhile the ULK1 –| AMPK–| ULK1 double negative feedback loop is able to “amplify” it. It is well known that this network motif is essential for a powerful mechanism for generating bistability and oscillation in a control network [19].

Although mTORC1 is a well-known autophagy inhibitor under physiological conditions the mTORC1 –| AMPK–| ULK1 and mTORC1 –| ULK1 –| AMPK regulatory connections assume that mTORC1 might have positive effects on autophagy induction. Recently Ganley et al. has shown that mTORC1 helps to maintain lysosome identity by promoting autophagic lysosome reformation (ALR). ALR-induced lysosomal tubulation is essential for the reformation of lysosomes from autolysosome upon long-term starvation. It has been confirmed that mTORC1 promotes both tubule initiation and tubule maintenance via phosphorylation of autophagy genes (such as UVRAG and VPS34) [20].

By doing computer simulations we suggest that the dynamical characteristic of autophagy induction gets periodically repeated both upon cellular stress (such as food deprivation) and in case of mTORC1 inhibition (mimicking e.g. rapamycin treatment) (Fig 1C). We hypothesize that the periodic activation-inactivation of ULK1 and AMPK under cellular stress or during mTORC1 inhibition explains the variance in experimental results found in the literature (such as ULK1 and AMPK gets activated or not during the given treatment [13, 21–25]) when the activity of key proteins was not monitored over time by multiple sampling, but only at a specific time point (see black dotted lines on Fig 1C, which could be our possible sampling points with different results).

Kazyken et al. also showed that AMPK promoted the reactivation of mTORC1, while being able to suppress ULK1-dependent autophagy induction in the presence of prolonged amino acid starvation [16]. We hypothesize that if amino acid starvation had been monitored for longer, mTORC1 would have been inactivated again and cell autophagy would have been induced. We have previously experimentally demonstrated by using multiple sampling points over a much longer time period that autophagy got an oscillatory characteristic both during glucose deprivation and during rapamycin treatment in HEK293 cells [26].

We suggest that the role of periodic repetitive autophagy in various cellular stresses is to allow the cell to use up damaged or unnecessary components generated during autophagy-dependent digestion. We claim that over time the amplitude of the autophagy oscillation decays and if conditions do not improve, the cell will somehow commit suicide. Ganley et al. has shown that some reactivation of mTORC1 is required for ALR to occur and thus new autophagosomes to form for a subsequent round of autophagy [20], further supporting the hypothesis that delayed negative feedback loops in autophagy regulation helps cell survival by generating a periodic activation during prolonged starvation. Recently, Mukhopadhyay et al. has observed two peaks of autophagy induction upon serum starvation in various cell types (such as HeLa, MDA-MB-231 and FaDu cells) assuming the oscillatory characteristic of autophagy induction, however prolonged food deprivation resulted in autophagy-dependent cell death [27].

### The importance of ULK1 -| AMPK connection in the periodic repeat of autophagy induction

**Fig 2:**
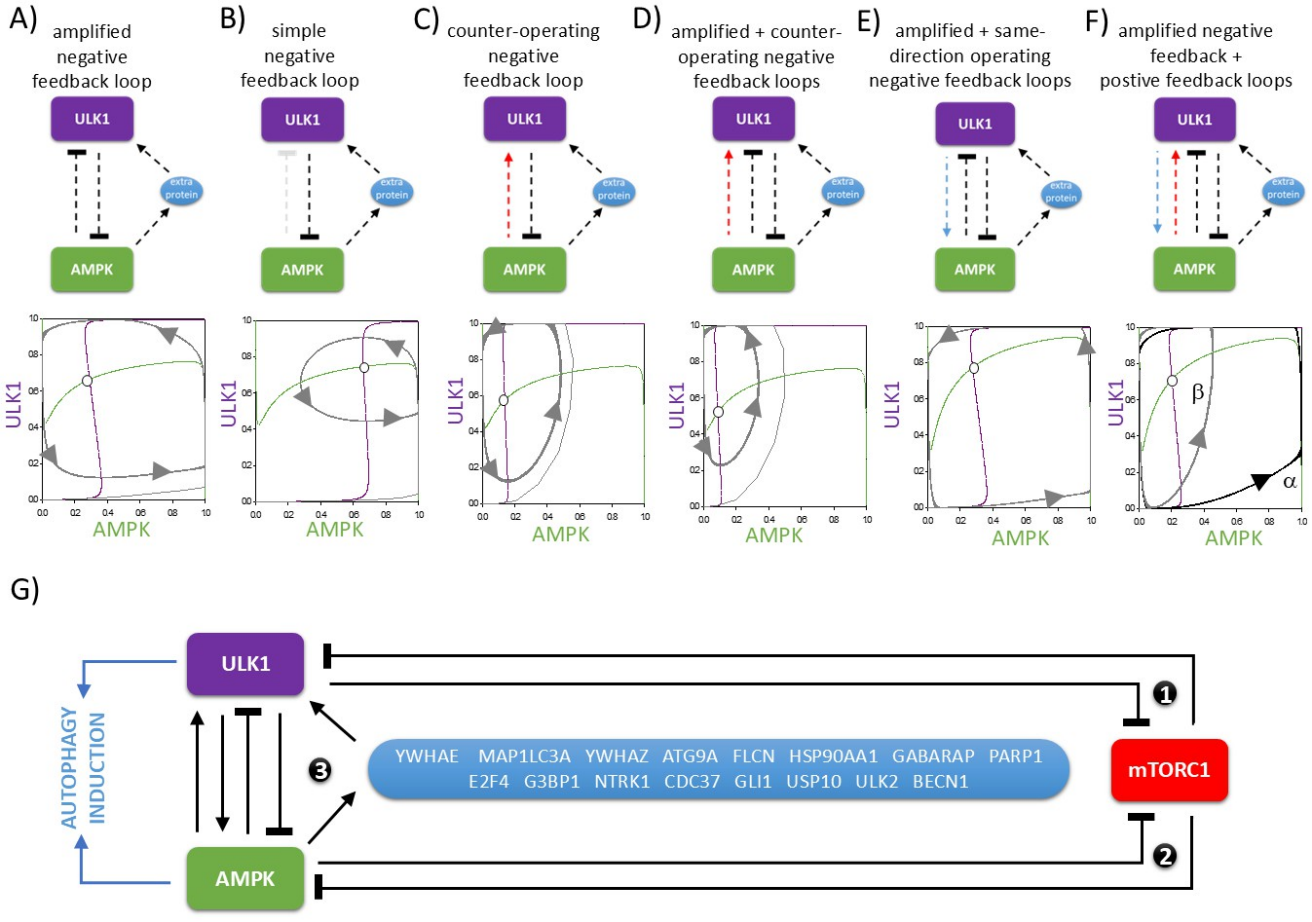
Confirming the role of the regulatory connections of autophagy induction. The (**upper panel**) simple wiring diagram of AMPK-ULK1 connection and the (**lower panel**) phase plane diagrams are plotted upon cellular stress when (**A**) amplified negative feedback loop, (**B**) simple feedback loop, (**C**) counter-operating negative feedback loop, (**D**) both amplified and counter-operating negative feedback loops, (**E**) amplified + same-direction operating negative feedback loops or (**F**) amplified negative feedback + positive feedback loops are present. On the wiring diagram dashed lines show how the molecules can influence each other, while locked end lines denote inhibition. On the phase plane diagram, the balance curves of ULK1 (green) and AMPK (orange) are plotted. Intersections of nullclines represent unstable (unfilled circle) steady state. Trajectories are depicted with dotted grey lines. (**G**) The wiring diagram of autophagy induction upon cellular stress completed with the possible ‘extra proteins’. Dashed lines show how the molecules can influence each other. Blocked end lines denote inhibition. Numbers refer to the negative and double negative feedback loops of the control network.

To further confirm the importance of amplified negative feedback loop between AMPK and ULK1, we plot the phase plane diagram of AMPK and ULK1 nullclines (Fig 2A–2F). The AMPK nullcline (green curve) and ULK1 nullcline (purple curve) refers to those points where the rate of activation is exactly balanced by the rate of inactivation, respectively. Along the AMPK nullcline, dAMPK/dt = 0 and trajectories move horizontally (i.e. there is no change in in the X direction but there may be change in the Y direction), meanwhile the ULK1 nullcline, dULK1/dt = 1 and trajectories move vertically (i.e. there is no change in the Y direction but there may be change in the X direction). By definition, when the nullclines intersect the system might have real biological states, which can be stable or unstable.

In the case where a delayed negative feedback loop between AMPK and ULK1 is complemented by a double negative feedback loop, the nullclines have one unstable intersection, which around the control network repeatedly overshoot and undershoot (see the grey trajectories on Fig 2A) generating a sustained oscillation of ULK1 and AMPK activity. The wider the trajectories run on the phase diagram, the more it is ensured that ULK1 and AMPK are properly turned on and off during oscillation.

If the double negative feedback loop between AMPK and ULK1 is removed, we assume only a simple delayed negative feedback loop (light grey dotted connection represents the absence of AMPK -| ULK1 connection form the wiring diagram on Fig 2B) in the regulatory network. In this case autophagy induction still has its oscillatory characteristic, but the amplitude of periodic repeat of both AMPK and ULK1 gets drastically reduced (Fig 2B). If the network motif is extended with a direct AMPK -> ULK1 regulatory connection (see red dotted arrow on Fig 2C) generating a counter-operating negative feedback loop in the control network the amplitude of autophagy oscillation gets decreased. Although some experimental data have suggested that AMPK is directly promotes the activation of ULK1 via phosphorylation, our theoretical analysis suggest that the presence of the amplified feedback loop cannot stabilize the oscillation of AMPK and ULK1 even if the counter-operating feedback loop is also present in the control network (Fig 2D).

It is much better for the dynamic behavior of the regulator system if AMPK does not act as an activator of the ULK1, but vice versa, i.e. ULK1 -> AMPK is combined with the amplified negative feedback loop (Fig 2E). In this case a sustained oscillation of autophagy is generated with the widest amplitude of AMPK and ULK1 during the cycles. This is entirely consistent with those very recent experimental results, where Yanagi et al. suggests that ULK1 can directly promote AMP sensitivity of AMPK by phosphorylation of AMPKγ1 at Ser260/Thr262 [28, 29]. Besides, the ULK1 -> AMPK link, which has been experimentally confirmed for many years, is also present in the regulatory system, but it is indirectly via the ULK1 –| mTOR–| AMPK pathway. It is important to note that when the ULK -> AMPK connection is combined with the AMPK -> ULK1 connection, a positive feedback loop is created. If we add this feedback loop to our amplified negative feedback loop we can get two results, depending on how strong the AMPK -> ULK1 connection is (Fig 2F). While a weaker ULK1 -> AMPK connection can increase the amplitude of autophagy oscillation (see nullclines and grey trajectories labelled “α” on Fig 2F), a larger value drastically decreases it (see black trajectories labelled “β” on Fig 2F).

We theorize that the dynamic behavior of the system is the most favorable when both amplified negative feedback loop and a positive feedback loop are present in the control system (Fig 2F). We assume that this scenario is the best for the control network to generate a sustained oscillation of autophagy with the widest amplitude and with the more robust answer, but this needs to be experimentally verified in the near future.

### Confirming the sign of regulatory components using bioinformatics methods

We hypothesize that not only AMPK and ULK1 fit into the above-mentioned regulatory network, but that other regulatory components with the same connections and their corresponding sign (such as positive or negative) may also play a role in the regulation of autophagy induction. All proteins that follow AMPK kinetics were named autophagy inducers, while components with ULK1 characteristics were named autophagy controllers (Fig 2G). This was followed by a systematic search for potential candidates using AutophagyNet—a network database of autophagy regulation [18]. By collecting protein-protein interactions from the core, direct regulator and further regulators layers, we defined a set of proteins that satisfy the requirements for the ‘extra protein’. Our results show that 16 proteins could fit the role of the ‘extra protein’ (Fig 2G).

## Conclusions

As we can see here the oscillatory characteristic of autophagy induction is crucial for the proper cellular response to stimuli. Taking into consideration newly available experimental data, our theoretical analysis has revealed that the presence of both amplified negative and positive feedback loops are essential to guarantee the periodic repeat of autophagy induction upon various cellular stresses. We argue that understanding the dynamical behavior of a process is greatly supported by approaching it from a systems biology perspective, and thus this analysis can greatly contribute to a much more precise study of the process experimentally. Therefore, the results presented here can greatly contribute to a more thorough understanding of how autophagy works.

## Supporting information

S1 Table Collection of data from literature on proven regulatory links in the ULK1-AMPK-mTORC1 regulatory network. Arrows indicate activation, while blocked end lines indicate inhibition between the members (ULK1, AMPK, mTORC1 and “autophagy”) of the network. “Autophagy” refers to the process by which autophagy actually takes place successfully in the cell. The asterisks refer to direct phosphorylation at a given site on the controlled protein. The numbers in the black boxes show the numbered connections in the regulatory network on Fig 1A [13, 15–17, 21, 28–62].
(XLSX)

S1 File (DOCX)

## Abbreviations

AMPK: 5′ adenosine monophosphate-activated protein kinase
mTORC1: mammalian target of rapamycin complex 1
ULK1: unc-51-like kinase 1
