# Supplementary material for: Oscillatory autophagy induction is enabled by an updated AMPK-ULK1 regulatory wiring
Source: PLoS One. 2024 Dec 26;19(12):e0313302. doi: 10.1371/journal.pone.0313302 (PMC11671020; doi:10.1371/journal.pone.0313302)
Supplement: S1 File — (DOCX) [file pone.0313302.s002.docx]

**Appendix A**

**Mathematical model used for computer simulations:**

Differential equation:

$$\frac{ULK1}{dt}=\frac{\left( kaulk+kaulk^{'}*Prot+kaulk"* AMPK \right)*(ULK1T-ULK1)}{Julk+ULK1T-ULK1}- \frac{(kiulk+kiulk^{'}*mTORC1+kiulk"*AMPK)*ULK1}{Julk+ULK1}$$

$$\frac{AMPK}{dt}=\frac{\left( kaak+kaak'*ULK1+stress1 \right)*(AMPKT-AMPK)}{Jampk+AMPKT-AMPK}- \frac{(kiak+kiak^{'}*ULK1+kiak"*mTORC1)*AMPK}{Jampk+AMPK}$$

$$\frac{ATG}{dt}=\frac{\left( kaau+kaau'*ULK1+kaau"*AMPK \right)*(1-ATG)}{Jatg+1-ATG}- \frac{(kiau+kiau^{'}*mTORC1)*ATG}{Jatg+ATG}$$

Steady state function:

$$mTOR=\frac{\left( kamtor*mTORC1T \right)}{kamtor+kimtor+kimtor^{'}*AMPK+kimtor"\text{*ULK1+kimtor"'}*Prot+stress2}$$

$$Prot=ProT*GK(kapr+kapr^{'}*AMPK,kipr,Jpr,Jpr)$$

'Goldbeter-Koshand' function (GK):

$$GB\left( arg1,arg2,arg3,arg4 \right)=arg2-arg1+arg2*arg3+arg1*arg4$$

$$GK\left( arg1,arg2,arg3,arg4 \right)=\frac{2*arg1*arg4}{(GB\left( arg1,arg2,arg3,arg4 \right)+sqrt\left( GB\left( arg1,arg2,arg3,arg4 \right)^{2}-4*\left( arg2-arg1 \right)*arg1*arg4 \right))}$$

**Table A1**

**The detailed description of the elements of the theoretical models and their value in physiological conditions.**

| Elements of the Theoretical Models | Description | Parameter Value |
| --- | --- | --- |
| ULK1 | The active form of ULK1 | Variable |
| ULK1T | Total level of ULK1 | 1 |
| AMPK | The active form of AMPK | Variable |
| AMPKT | Total level of AMPK | 1 |
| mTOR | The active form of mTORC1 | Variable |
| mTORT | Total level of mTORC1 | 1 |
| Prot | The active form of Prot | Variable |
| ProT | Total level of Prot | 1 |
| ATG | The active autophagy activator complex | Variable |

**Table A2**

**The detailed description of the constants of the theoretical models and their value in physiological conditions.**

| Constants of the Theoretical Models | Description | Parameter Value |
| --- | --- | --- |
| stress1 | stress acting on AMPK | 0.25 |
| stress2 | stress acting on mTOR | 0.75 |
| kaulk | Background activation of ULK1 | 1 |
| kaulk’ | Prot-dependent activation of ULK1 | 20 |
| kaulk” | AMPK-dependent activation of ULK1 | 0 |
| kiulk | Background inactivation of ULK1 | 0.25 |
| kiulk’ | mTOR-dependent inactivation of ULK1 | 10 |
| kiulk” | AMPK-dependent inactivation of ULK1 | 0.5 |
| Julk | Michaelis constant o ULK1 | 0.001 |
| kaak | Background activation of AMPK | 1.5 |
| kaak’ | ULK1-dependent activation of AMPK | 1 |
| kiak | Background inactivation of AMPK | 0.1 |
| kiak’ | ULK1-dependent inactivation of AMPK | 2.5 |
| kiak” | mTOR-dependent inactivation of AMPK | 7.5 |
| Jampk | Michaelis constant of AMPK | 0.001 |
| kamtor | Background activation of mTORC1 | 0.25 |
| kimtor | Background inactivation of mTORC1 | 0.05 |
| kimtor’ | AMPK-dependent inactivation of mTORC1 | 7.5 |
| kimtor” | ULK1-dependent inactivation of mTORC1 | 0.75 |
| kimtor’” | Prot-dependent inactivation of mTORC1 | 0 |
| kapr | Background activation of Prot | 0.01 |
| kapr’ | AMPK-dependent activation of Prot | 3 |
| kipr | Background inactivation of Prot | 30 |
| Jpr | Michaelis constant of Prot | 0.1 |
| kaau | Background activation of ATG | 0.01 |
| kaau’ | ULK1-dependent activation of ATG | 20 |
| kaau” | AMPK-dependent activation of ATG | 2 |
| kiau | Background inactivation of ATG | 0.1 |
| kiau’ | mTORC1-dependent inactivation of ATG | 100 |
| Jatg | Michaelis constant of ATG | 0.01 |

**Appendix B**

**Mathematical model for simulating signal–response curves:**

Differential equation:

$$\frac{ULK1}{dt}=\frac{\left( kaulk+kaulk^{'}*Prot+kaulk"* AMPK \right)*(ULK1T-ULK1)}{Julk+ULK1T-ULK1}- \frac{(kiulk+kiulk^{'}*mTORC1+kiulk"*AMPK)*ULK1}{Julk+ULK1}$$

$$\frac{AMPK}{dt}=\frac{\left( kaak+kaak'*ULK1+stress1 \right)*(AMPKT-AMPK)}{Jampk+AMPKT-AMPK}- \frac{(kiak+kiak^{'}*ULK1+kiak"*mTORC1)*AMPK}{Jampk+AMPK}$$

Steady state function:

$$mTORC1=\frac{\left( kamtor*mTORC1T \right)}{kamtor+kimtor+kimtor^{'}*AMPK+kimtor"\text{*ULK1+kimtor"'}*Prot+stress2}$$

$$Prot=ProT*GK(kapr+kapr^{'}*AMPK,kipr,Jpr,Jpr)$$

'Goldbeter-Koshand' function (GK):

$$GB\left( arg1,arg2,arg3,arg4 \right)=arg2-arg1+arg2*arg3+arg1*arg4$$

$$GK\left( arg1,arg2,arg3,arg4 \right)=\frac{2*arg1*arg4}{(GB\left( arg1,arg2,arg3,arg4 \right)+sqrt\left( GB\left( arg1,arg2,arg3,arg4 \right)^{2}-4*\left( arg2-arg1 \right)*arg1*arg4 \right))}$$

**Table B1**

**The detailed description of the elements of the theoretical models and their value in physiological conditions:**

| Elements of the Theoretical Models | Description | Parameter Value |
| --- | --- | --- |
| ULK1 | The active form of ULK1 | Variable |
| ULK1T | Total level of ULK1 | 1 |
| AMPK | The active form of AMPK | Variable |
| AMPKT | Total level of AMPK | 1 |
| mTORC1 | The active form of mTORC1 | Variable |
| mTORC1T | Total level of mTORC1 | 1 |
| Prot | The active form of Prot | Variable |
| ProT | Total level of Prot | 1 |

**Table B2**

**The detailed description of the constants of the theoretical models and their value in physiological conditions.**

| Constants of the Theoretical Models | Description | Parameter Value |
| --- | --- | --- |
| stress1 | stress acting on AMPK | 0.25 |
| stress2 | stress acting on mTOR | 0.75 |
| kaulk | Background activation of ULK1 | 1 |
| kaulk’ | Prot-dependent activation of ULK1 | 20 |
| kaulk” | AMPK-dependent activation of ULK1 | 0.25 |
| kiulk | Background inactivation of ULK1 | 0.25 |
| kiulk’ | mTOR-dependent inactivation of ULK1 | 10 |
| kiulk” | AMPK-dependent inactivation of ULK1 | 0.5 |
| Julk | Michaelis constant o ULK1 | 0.001 |
| kaak | Background activation of AMPK | 1.5 |
| kaak’ | ULK1-dependent activation of AMPK | 1 |
| kiak | Background inactivation of AMPK | 0.1 |
| kiak’ | ULK1-dependent inactivation of AMPK | 2.5 |
| kiak” | mTOR-dependent inactivation of AMPK | 7.5 |
| Jampk | Michaelis constant of AMPK | 0.001 |
| kamtor | Background activation of mTORC1 | 0.25 |
| kimtor | Background inactivation of mTORC1 | 0.05 |
| kimtor’ | AMPK-dependent inactivation of mTORC1 | 7.5 |
| kimtor” | ULK1-dependent inactivation of mTORC1 | 0.85 |
| kimtor’” | Prot-dependent inactivation of mTORC1 | 0 |
| kapr | Background activation of Prot | 0.01 |
| kapr’ | AMPK-dependent activation of Prot | 3 |
| kipr | Background inactivation of Prot | 30 |
| Jpr | Michaelis constant of Prot | 0.1 |
